# Preparation of TiSi_2_ Powders with Enhanced Lithium-Ion Storage via Chemical Oven Self-Propagating High-Temperature Synthesis

**DOI:** 10.3390/nano11092279

**Published:** 2021-09-02

**Authors:** Jianguang Xu, Menglan Jin, Xinlu Shi, Qiuyu Li, Chengqiang Gan, Wei Yao

**Affiliations:** School of Materials Science and Engineering, Yancheng Institute of Technology, Yancheng 224051, China; Jinml1234567@163.com (M.J.); Shixinlu1998@163.com (X.S.); liqiuyu945@163.com (Q.L.); 15905103523@163.com (C.G.)

**Keywords:** TiSi_2_, self-propagating high-temperature synthesis, electrochemical performance, lithium-ion battery

## Abstract

Although silicon has highest specific capacity as anode for lithium-ion battery (LIB), its large volume change during the charge/discharge process becomes a great inevitable hindrance before commercialization. Metal silicides may be an alternative choice because they have the ability to accommodate the volume change by dispersing Si in the metal matrix as well as very good electrical conductivity. Herein we report on the suitability of lithium-ion uptake in C54 TiSi_2_ prepared by the “chemical oven” self-propagating high-temperature synthesis from the element reactants, which was known as an inactive metal silicide in lithium-ion storage previously. After being wrapped by graphene, the agglomeration of TiSi_2_ particles has been efficiently prevented, resulting in an enhanced lithium-ion storage performance when using as an anode for LIB. The as-received TiSi_2_/RGO hybrid exhibits considerable activities in the reversible lithiation and delithiation process, showing a high reversible capacity of 358 mAh/g at a current density of 50 mA/g. Specially, both TiSi_2_ and TiSi_2_/RGO electrodes show a remarkable enhanced electrochemical performance along with the cycle number, indicating the promising potential in lithium-ion storage of this silicide. Ex-situ XRD during charge/discharge process reveals alloying reaction may contribute to the capacity of TiSi_2_. This work suggests that TiSi_2_ and other inactive transition metal silicides are potential promising anode materials for Li-ion battery and capacitor.

## 1. Introduction

The fast development of portable electrical equipment and electric vehicle creates a daunting demand for energy storage devices with high energy and power densities [1]. As a key unit for energy storage, lithium-ion batteries are experiencing a revolutionary tendency to reach the target of lightweight, long lifetime and good cycleability. In considering the low capacity (372 mAh/g) of commercial carbon anode, great efforts have been done to find some novel anode materials with high capacity. Due to the extremely high theoretical capacity (4200 mAh/g), silicon becomes one of the most attractive anode materials for lithium-ion battery [2,3,4]. However, it sustains from its low intrinsic electric conductivity and large structural volume changes during charge/discharge cycling [5]. This gives rise to serious mechanical stresses that consequently results in the pulverization of the active mass particles, eventually inevitable capacity fade [6,7,8].

As an attempt to overcome the drawbacks of silicon, metal silicides, which have good electric conductivity, were first investigated by Anani and Huggins as anodes for lithium-ion battery [9]. Since then, more researches have been done to evaluate the electrochemical behavior of metal silicides [10,11]. For example, the specific capacity of Mg_2_Si with an anti-fluorine structure is as high as ~400 mAh/g [12,13], and after being coated with carbon, the capacity of C@Mg_2_Si further reached 726 mAh/g [14]. The CrSi_2_ and MoSi_2_ synthesized by high energy ball milling show a reversible capacity of 340 and 130 mAh/g after 20 cycles at C/12, respectively, which is much higher than the commercial counterparts [15]. In the case of TiSi_2_, although the ordinary C54 type was reported as an inactive anode material for LIB [16], the C49 type nanonet shows excellent electrochemical performance as an anode, the reversible specific capacity of which is greater than 500 mAh/g [17]. Unfortunately, the application of this TiSi_2_ nanonet may hindered by the high cost of CVD method using SiH_4_ and TiCl_4_ as raw materials. Compared to the metastable C49 TiSi_2_, the C54 TiSi_2_ is thermodynamic stable and easier to be mass produced via cost effective method. In addition, if C54 TiSi_2_ could be transformed to low silicon silicides such as TiSi or Ti_5_Si_3_ during lithiation, it will exhibit high lithium-ion storage potential.

Thus, considering the low cost and remarkable potential in lithium-ion storage, we decided to explore the electrochemical performance of C54 TiSi_2_ as an anode for LIB. TiSi_2_ has been found to have broad applications as a thin film in microelectronics due to its remarkably high electrical conductivity, high temperature stability, and excellent mechanical property and corrosion resistance [18,19,20]. In addition, TiSi_2_ improved the battery behavior of Si-based anode materials significantly through increasing the electrical conductivity when it has been added to Si-based anode [21,22,23]. Specially, the TiSi_2_-air system exhibits very high energy density, which is more than 3–10 times higher than that of zinc-air or aluminum-air systems [24]. However, although Anani and Huggins predicted that TiSi is a potential anode material for LIB [9], the bulk C54 TiSi_2_ shows negligible capacity as we discussed above [16]. Fortunately, it has been well accepted that, the nanosized materials will show enhanced properties with respect to their corresponding bulk counterpart [25,26,27]. The reversible capacity of TiSi_2_ nanowire is better than the bulk one, although its value is still very low (65 mAh/g) [17]. On the other hand, designing special structure, including doping, making defects and adding carbon, is considered as an alternate effective way for optimizing the activity of the electrode. For example, The specific capacity of graphene was detected to be 540 mAh/g [28], while that of nitrogen-doped graphene achieved 684 mAh/g [29], and the flash reduced graphene with many defects have the ability of tolerating high current rate (156 mAh/g at 40C) [30]. The capacity of the interconnected porous silicon/carbon hybrids is kept at 1036.6 mAh/g after 100 cycles at 50 mA/g [31], and the graphene/Si–C hybrid anode shows high areal capacity (3.2 mAh/cm^2^ after 100 cycles at 50 mA/g) for LIB due to its dual conductive network [32].

In this work, pure TiSi_2_ was synthesized by the “chemical-oven” self-propagating high-temperature synthesis (COSHS). The merits of SHS lie on the fast reaction, energy-saving, and the ability to form very fine particles with many defects if considering high-temperature deformation and fast transformation [33,34]. Then, reduced graphene oxide (RGO) was used to wrap up the TiSi_2_ particles for constructing a homogeneous hybrid with high conductive network. The battery performance of the TiSi_2_ and TiSi_2_/RGO anodes were investigated in detail.

## 2. Materials and Methods

### 2.1. Synthesis of TiSi_2_

99% pure titanium powder with an average particle size of 35 μm, and 99% pure Si powder with an average particle size of 65 μm were used in this work. The mixture of Si and Ti (atomic ratio 2:1) was immersed in ethanol in a steel container and then ball milled using agate balls at a speed of 300 rpm for 3 h. The resulting slurry was dried in a drying cabinet at 80 °C for 5 h. The dried powder was then sieved to 100 mesh and collected for the following “chemical oven” self-propagating high-temperature synthesis (COSHS) procedure. The Ti + 2Si mixture was placed inside a graphite crucible, while the Ti + C mixture, used as a chemical furnace, was placed outside. Carbon is present between the inside and outside. The two mixtures are in contact with each other at the bottom of the graphite crucible. Then, the powders were burned via electrifying tungsten placed in the upper surface of the outer powders in a steel chamber at a pressure of 0.2 MPa Ar (99.9 wt.%). The synthesized powder was collected and ball milled at a speed of 300 rpm for 3 h to perform the following operations.

In order to remove impurities in the synthesized TiSi_2_ powder, 3 g of TiSi_2_ powder was dispersed in 100 mL of 2 M NaOH aqueous solution and stirred vigorously for 24 h. Then the purified TiSi_2_ was collected via washing the mixture in deionized water and vacuum drying.

### 2.2. Synthesis of Graphite Oxide

Graphite oxide (GO) was synthesized by a modified Hummers method. Generally, a mixture of 3 g graphite, 2.5 g P_2_O_5_, 2.5 g K_2_S_2_O_8_ and 12 mL concentrated H_2_SO_4_ was stirring for 6 h in a flask at 80 °C. After cooling, it was diluted carefully with deionized water, then filtered and washed on the filter until the pH value of the washed water became neutral. After drying at the ambient temperature, the obtained pre-oxidized graphite powder was dispersed in 120 mL of concentrated H_2_SO_4_ in an ice bath. Then, 30 g of KMnO_4_ was added to the dispersion gradually with stirring. After that, the mixture was stirred at 35 °C for 2 h, and then 200 mL of deionized water was added dropwise. The dispersion was then stirred for another 30 min, and the reaction was stopped by adding 0.5 L of deionized water and 20 mL of 30% H_2_O_2_, after which the color of the dispersion changed to bright yellow. The dispersion was filtered and washed with 1 M HCl aqueous solution to remove metal ions. For further purification, graphitate oxide was dialyzed weekly to remove residual salts and acids. The concentration of the resulting solution was ~7 mg/g.

### 2.3. Preparation of TiSi_2_/Graphene (RGO) Hybrid Material

The hybrid material was synthesized based on a simple one-pot method. Usually, 3.05 g of graphite oxide solution was dispersed in 100 mL of deionized water and ultrasonically treated for 1 h, and then left to stand in a magnetic stirrer for 6 h to produce GO solution. After that, 0.103 g of purified TiSi_2_ powder was added to the solution, followed by ultrasonic treatment for 1 h and stirring for 6 h. Then, 30 μL of 80% hydrazine was added to the mixed solution and stirred in a water bath at 30 °C for 12 h. Finally, the precipitate was gathered via centrifugation and washing with ethanol and deionized water.

### 2.4. Preparation of Electrodes

The CR-2032 coin cells using lithium metal film as the counter electrode and reference electrode were used to evaluate the electrochemical performance of TiSi_2_ particles and TiSi_2_/graphene hybrid particles. The battery is based on lithium metal (−)|electrolyte|TiSi_2_ or TiSi_2_/RGO hybrid (+), and the electrolyte is 1 M LiPF_6_ in ethylene carbonate (EC)-dimethyl carbonate (DMC)-methyl ethyl carbonate (EMC) solution (volumetric ratio 1:1:1). A microporous polypropylene film (Celgard 2400, Shanghai, China) was used as the separator. 80 wt.% TiSi_2_ or TiSi_2_/RGO with 10 wt.% acetylene black and 10 wt.% polyvinylidene fluoride (PVDF) binder were mixed uniformly in N-methyl pyrrolidinone (NMP), resulting into a viscous slurry for effective deposition. To prepare electrode, the as-received slurry was deposited on a copper foil (10 μm) current collector and dried in a vacuum oven at 100 °C for 12 h. The mass loading of active material is 0.65–0.85 mg/cm^2^. Cell assembly was conducted in a glove box filled with pure argon.

### 2.5. Material Characterization

The morphologies of TiSi_2_ and TiSi_2_/RGO hybrid were investigated by a scanning electron microscope. The phase structure was determined by X-ray diffraction analysis of Cu-Kα radiation (λ = 1.5418 Å) at room temperature in the 2θ range of 20° to 70°. FT-IR spectroscopy was performed with a NEXUS-670 Fourier Transform Infrared Spectrometer (Ramsey, MN, USA). The surface area and pore size distribution were measured on a TriStar II 3020 (Norcross, GA, USA) surface area and porosity analysis instrument.

The cells were galvanostatically charged and discharged within a constant voltage range of 0.001 to 3 V on the Shenzhen Neware battery cycler (Shenzhen, China) at room temperature. All gravimetric capacity corresponding to the prepared electrodes were calculated based on the mass of TiSi_2_ or TiSi_2_/RGO hybrid. The electrochemical workstation (Zahner-Zennium, Kronach, Germany) was used for cyclic voltammetry and electrochemical impedance spectroscopy (EIS, frequency range: 0.001–10^5^ Hz, amplitude: 5 mV) analysis. In this work, unless otherwise specified, all impedance measurements were performed after three cycles of the prepared electrodes.

## 3. Results and Discussion

**Figure 1:**
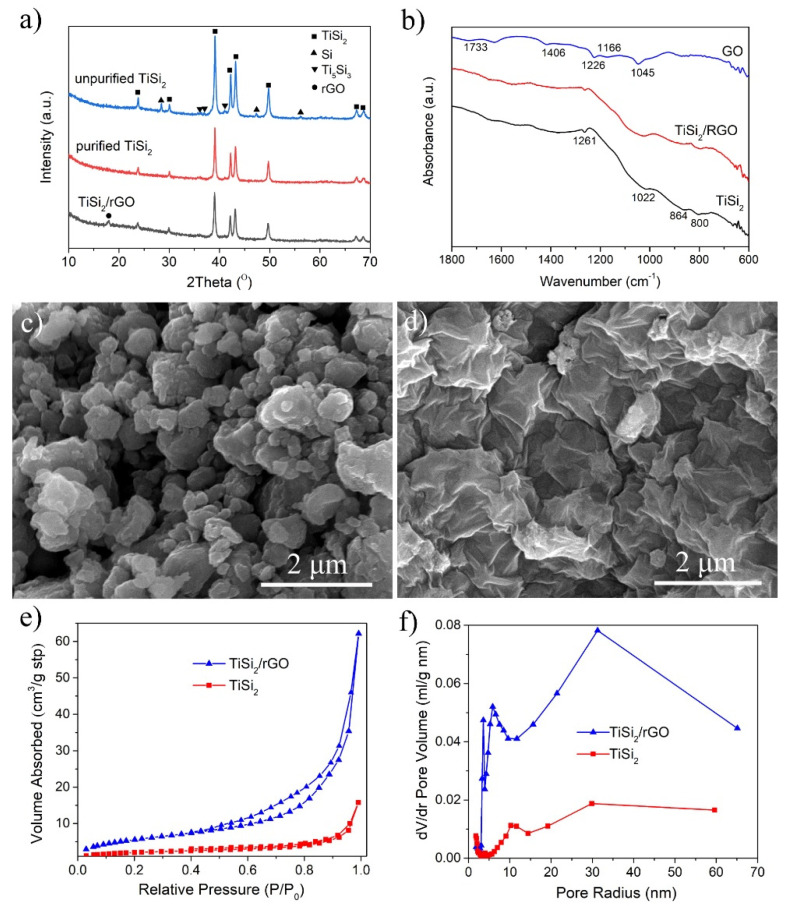
(**a**) XRD patterns of TiSi_2_, purified TiSi_2_ and TiSi_2_/RGO; (**b**) FT-IR spectra of graphene oxide (GO), TiSi_2_ and TiSi_2_/RGO; SEM micrographs of (**c**) TiSi_2_ and (**d**) TiSi_2_/RGO; (**e**) N_2_ adsorption-desorption isotherms and (**f**) pore size distributions of TiSi_2_ and TiSi_2_/rGO.

The XRD pattern of the combustion product (unpurified TiSi_2_) shows that TiSi_2_ was successfully synthesized by the chemical oven SHS method. There are no other titanium silicides phases except TiSi_2_ detected based on the XRD result. Because TiSi_2_ is the first phase generated in the formation sequence among all the possible titanium silicides [35], when using Ti + 2Si mixture as raw material, TiSi_2_ is the only reaction product. Besides the main phase TiSi_2_, residual Si is also detected. It can be observed from the XRD pattern of purified TiSi_2_ that the residual Si was thoroughly removed after treating the product in NaOH aqueous solution. Figure 1a also exhibits the XRD pattern of TiSi_2_/RGO hybrid, indicating the crystal structure of TiSi_2_ has no change after hybridizing purified TiSi_2_ with RGO.

Meanwhile the TiSi_2_/RGO hybrid is further proved by FT-IR spectra. Figure 1b shows the FT-IR spectra of graphene oxide (GO), TiSi_2_, and TiSi_2_/RGO hybrid. As shown in Figure 1b, the pattern of GO exhibits a few peaks because there are some oxygen functional groups on its surface. The peak at 1733 cm^−1^ is related to the stretching vibration of –C=O/–COOH. The peaks at 1406 and 1226 cm^−1^ are corresponding to the stretching vibration of O-H and C–OH in tertiary alcohol, respectively, and those at 1166 and 1045 cm^−1^ are associated with the absorption of C–O [36]. These peaks disappeared when the GO has been reduced, thus they cannot be detected in the pattern of TiSi_2_/RGO hybrid. In addition, the FT-IR spectra of TiSi_2_ and TiSi_2_/RGO are similar, indicating the TiSi_2_/RGO was successfully obtained in this work and TiSi_2_ did not change during the reduction process. It can be also concluded from the FT-IR spectra that TiSi_2_ particles are coated by a thin oxide film, because the weak peaks at 1264, 1022, and 800 cm^−1^ can be attributed to Si–O–Si bond, while the peak at 864 cm^−1^ is corresponding to Si–O–Ti bond [37].

Figure 1c,d shows SEM micrographs of TiSi_2_ and the TiSi_2_/RGO hybrid. The as-synthesized TiSi_2_ particles range from approximately 0.3 μm to 2 μm, and each particle consists of many small primary particles. This aggregation of TiSi_2_ primary particles will prevent the diffusion of Li ion, resulting in a limited electrochemical performance. In this consideration, sonication was applied to avoid the agglomeration during hybridizing TiSi_2_ with graphene. As a result, separated TiSi_2_ particles have been warped up tightly by folded RGO nanosheets which were in situ reduced by hydrazine in the graphene oxide/TiSi_2_ hybrid dispersion, showing a good distribution in RGO nanosheets. In addition, RGO nanosheets contacted well with each other, resulting in a fast electron conductive network when using as an electrode for LIB.

The exposed surface area and pore size distributions of TiSi_2_ and TiSi_2_/RGO hybrid were further investigated by N_2_ absorption/desorption tests, shown Figure 1e,f. A typical type-IV isotherm curve of TiSi_2_/RGO with an obvious hysteresis loop of type H3 can be observed in Figure 1e, indicating the incorporation of TiSi_2_ particles between RGO nanosheets giving rise to slit-shaped pores. In addition, the surface area of TiSi_2_/RGO is 21.33 m^2^/g, increased by 205.6% compared to that of pure TiSi_2_. The increased surface area suggested that adding RGO nanosheets can effectively inhibit the aggregation and the in-situ hybridization of RGO nanosheets. Moreover, as shown in Figure 1f, the TiSi_2_/RGO hybrid possesses more pores than pure TiSi_2_. These results obtained by BET method confirmed the porous structure of TiSi_2_/RGO hybrid, which can supply channels for electrolyte transport and Li^+^ ions diffusion, leading to a better electrochemical performance.

**Figure 2:**
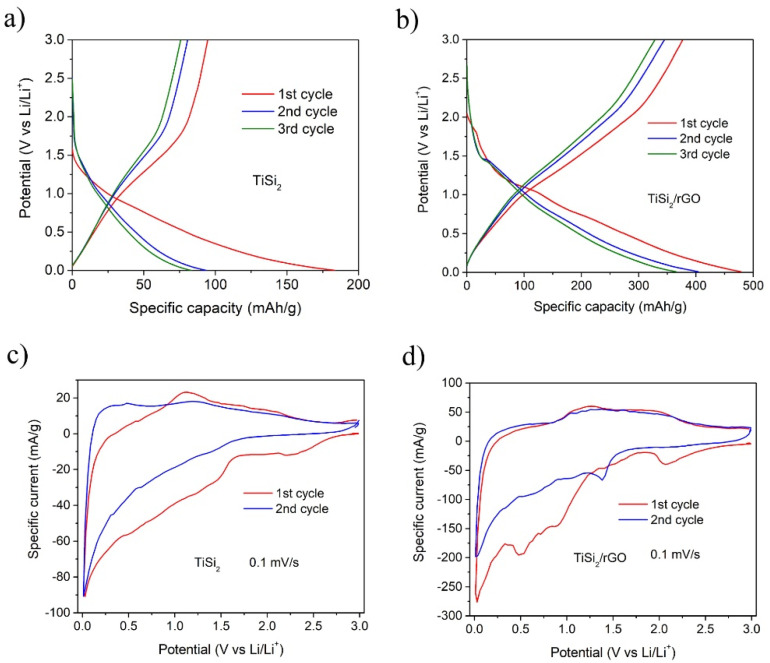
The first three voltage profiles at 50mA/g of (**a**) TiSi_2_ and (**b**) TiSi_2_/RGO; CV scans at 0.1 mV/s of (**c**) TiSi_2_ and (**d**) TiSi_2_/RGO.

Then, TiSi_2_ and TiSi_2_/RGO were investigated as anodes for LIBs. Figure 2a,b shows the first three charge-discharge profiles of the TiSi_2_ and TiSi_2_/RGO samples in the voltage range of 0.001 to 3 V at a current density of 50 mA/g. The initial charge and discharge capacities of TiSi_2_ electrode are 184 mAh/g and 95 mAh/g, respectively, and the relevant coulombic efficiency is 51.5%. However, for the graphene-modified TiSi_2_ anode, these specific capacities are increased to 478 mAh/g and 378 mAh/g, respectively, and the relevant coulombic efficiency is as high as 78.8%. Figure 2c,d shows the first two voltammograms (CVs) of TiSi_2_ and TiSi_2_/RGO hybrid at a scan rate of 0.1 mV/s. In the 1^st^ cathodic sweep, a peak at around 2.1 V in both anodes’ curves can be observed, corresponding to irreversible electrochemical reactions between surface silicon oxide and Li ion [38]. The broad peaks at 0.5–1.5 V for TiSi_2_ and 0.5–1.1 V for the hybrid can be assigned to the formation of SEI layer on TiSi_2_ particles. These peaks are unobserved during the following cycles. Then, a sharp peak close to 0 V appeared in both TiSi_2_ and the hybrid’s curves, which can be assigned to the formation of Li_x_Si [10,21,39]. Two consecutive anodic peaks can be discovered at 1.2 V and 2.0 V, which are probably the decomposition of Li_x_Si and formation of TiSi_2_. These peaks of the hybrid are still obvious in the second anodic sweep, while those of TiSi_2_ are very weak, indicating the electrochemical reactions in the hybrid are kept active during following charge/discharge cycles. Thus the hybrid exhibits better electrochemical performance compared to pure TiSi_2_.

**Figure 3:**
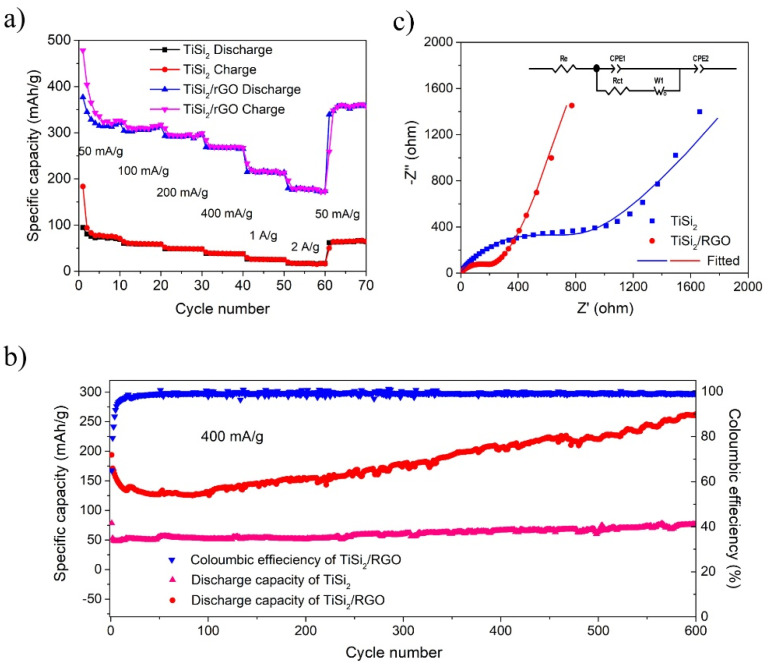
(**a**) rate performance of TiSi_2_ and TiSi_2_/RGO; (**b**) cycle performance and coulombic efficiency of TiSi_2_ and TiSi_2_/RGO at a current density of 400mA/gt; (**c**) Nyquist plots of TiSi_2_ and TiSi_2_/RGO.

Figure 3a shows the rate behavior of TiSi_2_ and TiSi_2_/RGO. When using TiSi_2_/RGO as an anode, a reversible capacity of 320 mAh/g has been achieved at a current density of 50 mA/g. When the current densities were increased to 100, 200, 400, 1000 and 2000 mA/g, the TiSi_2_/RGO anode delivered reversible capacities of 314, 296, 265, 214, and 174 mAh/g, respectively. Furthermore, when the current density was reverted to 50 mA/g, the TiSi_2_/RGO anode recovered a higher capacity of ~358 mAh/g, which is a ~112% of the initial specific capacity, exhibiting an excellent rate performance. However, the TiSi_2_ electrode has poor rate performance.

Figure 3b shows the cyclic performance of TiSi_2_ and TiSi_2_/RGO at a current density of 400 mA/g. The 1st discharge capacity of TiSi_2_/RGO is 194 mAh/g, while the 2nd discharge capacity is reduced to 171 mAh/g. Particularly, after a few initial cycles, the specific capacity started to gradually increase with cycles and a high specific capacity of 263 mAh/g has been reached after 600 charge-discharge cycles. Moreover, this value still fits a growing trend. The 1st coulombic efficiency is 65%, then it quickly increased to ~100% in a few cycles and maintained stable in following cycles. This lithium ions storage improvement with cycling can be attributed to the continuous activation of TiSi_2_ by repeated insertion of Li^+^, leading to much more electrochemically active sites for Li^+^ insertion or alloying. On the other hand, the pure TiSi_2_ anode only obtained a low specific capacity of 77 mAh/g after 600 charge-discharge cycles, which is much lower than the TiSi_2_/RGO anode. The low capacity of pure TiSi_2_ anode can be ascribed to the agglomeration of primary TiSi_2_ particles, preventing the electrolyte access and insertion of Li^+^.

In order to further investigate the lithium-ion storage property of these two anodes, electrochemical impedance spectroscopy (EIS) was employed at room temperature. It can be seen from Figure 3c—that both Nyquist plots are mainly composed of two parts: a semicircle in the high-frequency region represents the resistance at the Ohmic surface layer [40], and an oblique line in the low-frequency region indicates an ion diffusion procedure. The Nyquist plots were fitted using the modified Randles equivalent circuit (the inset in Figure 3c). Based on the fitting results, the TiSi_2_/RGO hybrid material exhibits a lower charge-transfer resistance of 209.6 Ω, while TiSi_2_ shows a higher transfer resistance of 920.0 Ω, suggesting the reduced graphene oxide improves the electronic conductivity significantly and TiSi_2_/RGO hybrid obtains a faster charge-transfer process as a result. This result is in a good agreement with the enhancement of electrochemical performance of TiSi_2_/graphene hybrid.

**Figure 4:**
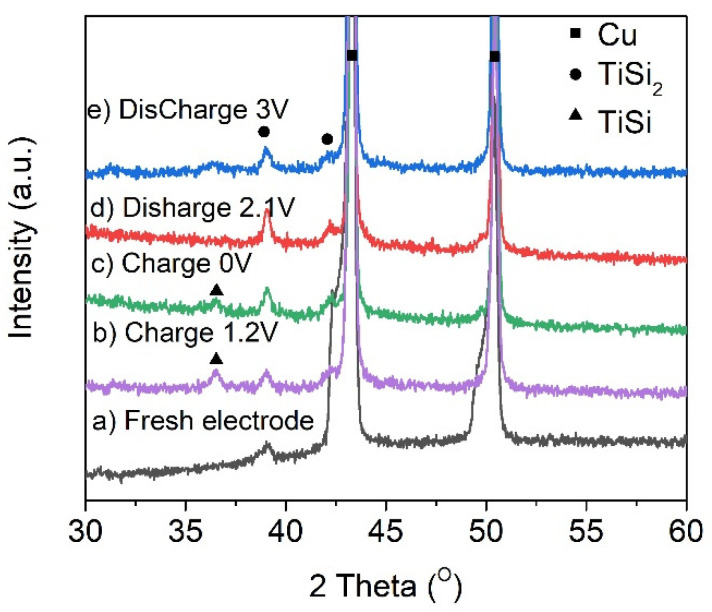
XRD patterns of TiSi_2_/RGO electrode at different charge/discharge potentials.

To confirm the detail of phase transformation during the lithiation/delithiation process, the TiSi_2_/RGO electrodes have been checked by XRD at different charge/discharge potentials (Figure 4). Compared with XRD pattern of the fresh electrode (Figure 4a), there is a new peak that appeared at ~37° in the XRD pattern when the electrode was charged to 1.2 V (Figure 4b) and 0 V (Figure 4c), indicating the formation of TiSi phase. Then this peak is disappeared when the electrode was discharged to 2.1 V (Figure 4d), which is probably due to the transformation from TiSi to TiSi_2_. According to the ex situ XRD results and in comparison with the reactions of MoSi_2_ and CrSi_2_ during the lithiation/delithiation process [15], TiSi_2_ would like to follow the reaction shown in Equation (1) [9]:

3TiSi_2_ + 7Li^+^ + 7e^−^ ←→ 3TiSi + 3SiLi_2.33_ (1)

This work further demonstrated that the Li-ion storage potential of inactive transition metal silicides is promising, as reported in our earlier work [41]. According to Equation (1), assuming TiSi_2_ can transform completely to TiSi and SiLi_2.33_, theoretical capacity values of 600 mAh/g can be expected for TiSi_2_. If TiSi could further decompose to Ti_5_Si_3_ or Ti_3_Si, this would increase the capacities to 840 or 1000 mAh/g, respectively. These capacity values are much higher than the initial reversible capacity of TiSi_2_ and TiSi_2_/RGO electrodes obtained in this work. However, with the charge-discharge cycles, a reversible capacity of 263 mAh/g at 400 mA/g was achieved for TiSi_2_/RGO hybrid, increased by 51% compared to the 2nd charge capacity, suggesting that increasingly more TiSi_2_ grains are decomposed with cycling. Thus it is rational to predict that the electrochemical performance of TiSi_2_ can be further remarkably enhanced by decreasing the particle size and engineering the materials’ structure and composition. In addition, compared to other inactive transition metal silicides (Table 1), the TiSi_2_/rGO electrode shows a moderate specific capacity and best capacity retention after cycles. Moreover, if using ionic liquid as electrolyte, this electrode may have a chance to achieve better electrochemical performance [42]. Combination of a relatively high capacity, low volume changes upon cycling and excellent rate capability of TiSi_2_/RGO hybrid, TiSi_2_ or other inactive transition metal silicides may become the next anode material to substitute graphite, silicon or lithium titanate, or at least act as high-rate electrode for Li-ion battery and capacitor.

## 4. Conclusions

In summary, the electrochemical performance of pure TiSi_2_ and TiSi_2_/RGO hybrid as anode materials for LIB was investigated in this work. Pure TiSi_2_ was obtained by the “chemical oven” self-propagating high-temperature synthesis from the element reactants. TiSi_2_/RGO hybrid was synthesized through reducing the graphene oxide/TiSi_2_ suspension by hydrazine. Benefiting from the high conductivity, the hybrid exhibits better activities than pure TiSi_2_ in the reversible lithiation and delithiation process, and its reversible specific capacity is ~263 mAh/g after 600 cycles at 400 mA/g, increased by 2.4 times compared to that of pure TiSi_2_. There are lots of inactive transition metal silicides with low activity at room temperature reported in literature, such as MoSi_2_, WSi_2_, and most of them have potentials such as good conductivity, good stability even under severe environments for using as an anode. Our discoveries are suggesting and encouraging the investigation on the inactive transition metal silicides as valuable electrodes as Li-ion battery and capacitor.

## Figures and Tables

**Table 1:** Comparison of electrochemical performance of different inactive transition metal silicides.

Materials	Electrolyte	Specific Capacity (mAh/g)	Capacity Retention/Cycles	Ref.
CrSi_2_	1 M LiFSA/Py13-FSA	240 (50 mA/g)	89%/300	[42]
FeSi_2_	1 M LiFSA/Py13-FSA	630 (50 mA/g)	85%/300	[42]
FeSi_2_	1 M LiTFSA/PC	700 (50 mA/g)	15%/300	[42]
NiSi_2_	1 M LiFSA/Py13-FSA	820 (50 mA/g)	58%/250	[42]
Nano-NiSi_2_	1 M LiPF_6_/EC-DC-FEC	313 (87.5 mA/g)	~88%/60	[43]
MoSi_2_	1 M LiPF_6_/EC-DMC-EMC	153 (134 mA/g)	127%/100	[41]
MoSi_2_	1 M LiPF_6_/EC-DMC	~110 (67 mA/g)	-	[15]
TiSi_2_/rGO	1 M LiPF_6_/EC-DMC-EMC	378 (50 mA/g)	136%/600	This work

## Data Availability

No new data were created or analyzed in this study. Data sharing is not applicable to this article.
